# Case Report: A rare *AGXT* pathogenic variant associated with young-adult-onset end-stage kidney disease

**DOI:** 10.3389/fgene.2026.1865152

**Published:** 2026-07-29

**Authors:** Hui Jiang, Fuzhen Wang, Senqing Lin, Senchao Wu, Xiuping Qiu, Jinxiu Deng

**Affiliations:** 1 Department of Nephrology, Longyan First Affiliated Hospital of Fujian Medical University, Longyan, China; 2 Department of Endocrinology, Longyan First Affiliated Hospital of Fujian Medical University, Longyan, China

**Keywords:** AGXT, blood purification, end-stage kidney disease, nephrolithiasis, plasma oxalate, primary hyperoxaluria type 1

## Abstract

Primary hyperoxaluria type 1 (PH1) is a rare autosomal recessive metabolic disorder caused by pathogenic variants in *AGXT*, leading to hepatic oxalate overproduction, recurrent nephrolithiasis, nephrocalcinosis, progressive kidney dysfunction, and, in advanced stages, systemic oxalosis. Here, we report a family in which two brothers developed recurrent nephrolithiasis and progressed to end-stage kidney disease (ESKD) in young adulthood. The proband, a 32-year-old man, presented with bilateral kidney stones, rapidly progressive renal failure, and extensive extra-renal tissue oxalate deposition on follow-up imaging. Genetic testing identified a homozygous *AGXT* variant, c.740T>G, p.(Leu247Arg), in both affected siblings, while the proband’s mother and daughter were heterozygous carriers, consistent with autosomal recessive inheritance. Plasma oxalate levels were markedly higher in the proband than in his brother and in non-PH ESKD controls. During follow-up, the proband received maintenance hemodialysis alone, whereas his older brother underwent combined hemodialysis, hemofiltration, and hemoperfusion, and appeared to achieve better plasma oxalate control. These findings suggest that intensified blood purification may serve as a potential bridging strategy in advanced PH1 while definitive treatment is being arranged. This report expands the genotypic and phenotypic spectrum of PH1 and underscores the importance of early genetic evaluation in patients with recurrent or early-onset nephrolithiasis, especially when accompanied by rapid renal decline or a positive family history.

## 1 Introduction

Primary hyperoxaluria (PH) is a group of rare autosomal recessive metabolic disorders characterized by excessive endogenous oxalate production in the liver, resulting in marked hyperoxaluria, recurrent calcium oxalate stone formation, nephrocalcinosis, and progressive kidney injury (Garrelfs et al., 2021a; Groothoff et al., 2023). PH type 1 (PH1), the most common form, is caused by pathogenic variants in *AGXT*, which encodes alanine: glyoxylate aminotransferase (AGT), a key enzyme in hepatic glyoxylate metabolism (Kang, 2024). Once kidney function declines substantially, renal oxalate excretion becomes insufficient, and systemic oxalate deposition may occur in bone, vessels, myocardium, nerves, skin, and eyes (Groothoff et al., 2023).

The clinical spectrum of PH1 is highly heterogeneous, ranging from infantile oxalosis to adult-onset recurrent nephrolithiasis (van Woerden et al., 2003; Dejban and Lieske, 2022). Because early manifestations often resemble common stone disease, the diagnosis is frequently delayed. Early recognition is clinically important because some patients may benefit from pyridoxine and newer RNA interference therapies targeting hepatic oxalate production (Hoppe and Martin-Higueras, 2022; Bahbah et al., 2025). However, once patients reach ESKD, standard dialysis is often insufficient to offset ongoing oxalate generation, and combined liver-kidney transplantation remains the only established curative treatment.

Here, we report a family with PH1 associated with a rare homozygous *AGXT* variant, c.740T>G, p.(Leu247Arg). Both affected siblings developed nephrolithiasis and ESKD in young adulthood. We also describe the clinical course of the proband and discuss the possible role of intensified blood purification as bridge therapy in advanced disease.

## 2 Case presentation

### 2.1 Patient information

The proband was a 32-year-old man admitted on 15 June 2018, because of elevated serum creatinine for more than 1 month. One month before admission, he presented to a local hospital with right flank pain. Laboratory evaluation showed a serum creatinine level of 716 μmol/L and a blood urea nitrogen level of 21.22 mmol/L. Urinary tract computed tomography (CT) demonstrated multiple bilateral renal calculi with hydronephrosis. He subsequently underwent ureteroscopic lithotripsy and stone extraction for a right ureteral stone, but renal function continued to deteriorate. At referral, serum creatinine had increased to 970 μmol/L, and the estimated glomerular filtration rate was 8.6 mL/min/1.73 m^2^.

His past medical history was notable for type 2 diabetes mellitus for 6 years, treated with oral hypoglycemic agents. Family history was significant in that his older brother had developed bilateral nephrolithiasis and uremia and had started hemodialysis at 27 years of age. Physical examination revealed bilateral blindness, marked tooth loss, painful subcutaneous nodules over both hips, and lower-limb dysfunction. Ophthalmological findings—including conjunctival hyperemia, vitreous haziness with spotty opacities, retinal dot/blot hemorrhages, and vascular wall abnormalities—are consistent with PH1-associated oxalate deposition in retinal capillaries and vitreous humor, leading to endothelial apoptosis and secondary neovascularization. Dental loss reflects oxalate-induced alveolar bone resorption, a recognized but underreported manifestation of systemic oxalosis distinct from periodontal disease in diabetes. These features were more severe in the proband (II-1) than his brother (II-2) (Table 1), correlating with higher plasma oxalate levels and extensive extra-skeletal calcification.

**TABLE 1:** Clinical, biochemical, and treatment characteristics of the two affected siblings.

Parameter	Proband (II-1)	Brother (II-2)
Age at ESKD onset, years	32	27
Blood pressure(mmHg)	142/89	130/60
Diabetes mellitus	Yes	No
eGFR, mL/min/1.73 m^2^	8.6	1.5
Plasma oxalate(μmol/L)	287.93	217.67
Ophthalmological findings	Retinal hemorrhages, vitreous opacities, and macular involvement	Not assessed
Dental status	Marked tooth loss	Intact dentition
Dialysis duration, h/week	10–12	12
Blood flow rate, mL/min	250	250–300
Dialysis modality	HD	HD + HDF + HP
Hemodialysis vascular access	Tunneled cuffed internal jugular venous catheter	Autogenous arteriovenous fistula

ESKD, end-stage kidney disease; eGFR, estimated glomerular filtration rate; HD, hemodialysis; HDF, hemodiafiltration; HP, hemoperfusion; II-1 denotes the proband, and II-2 denotes his brother. “Not available” indicates that the relevant test result was not provided in the case records; “Not assessed” indicates that the corresponding clinical examination was not performed.

### 2.2 Plasma oxalate quantification

Plasma oxalate concentrations were measured using a colorimetric oxalate assay kit based on oxalate oxidase chemistry, according to the manufacturer’s instructions (Sigma-Aldrich, MAK315). Briefly, 10 μL of plasma sample or oxalate standard was added to each well, followed by the working reagent containing the enzymatic color reaction components. After incubation for approximately 10 min at room temperature, absorbance was measured at 595 nm using a microplate reader. Oxalate concentrations were calculated from a standard curve generated using oxalate standards. The assay has a linear detection range of 20–1,500 μmol/L. Samples with concentrations exceeding the linear range were diluted and reanalyzed. Plasma oxalate concentrations were reported in μmol/L.

## 3 Clinical findings

Laboratory testing demonstrated severe renal insufficiency, with serum creatinine of 1,181 μmol/L and uric acid of 617 μmol/L, accompanied by metabolic acidosis (total carbon dioxide 12.8 mmol/L), hypocalcemia (1.82 mmol/L), hyperphosphatemia (2.27 mmol/L), anemia (hemoglobin 88 g/L), and elevated parathyroid hormone (247.4 ng/L). Imaging confirmed bilateral multiple renal stones and renal atrophy.

Historical clinical data from the proband’s older brother at 27 years of age showed an even more severe presentation, including serum creatinine of 2,884 μmol/L, hyperkalemia (6.5 mmol/L), severe anemia (hemoglobin 42 g/L), and cardiac dysfunction with brain natriuretic peptide >30,000 pg/mL.

## 4 Genetic findings and family segregation

**FIGURE 1:**
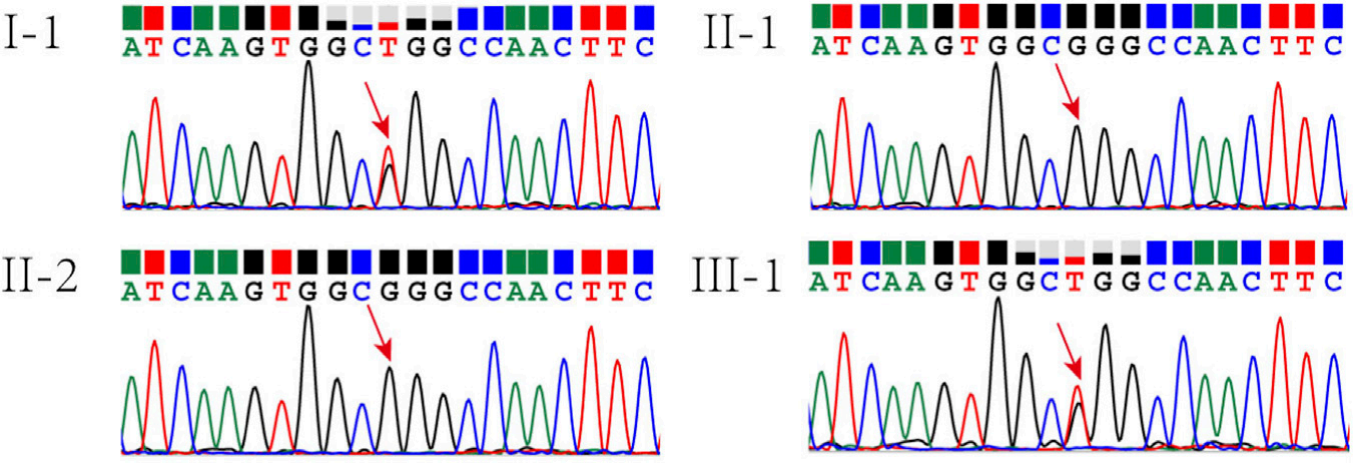
Sanger sequencing chromatograms of the target locus in family members. Representative electropherograms and corresponding sequences are shown for I-1 (mother of the proband), II-1 (proband), II-2 (brother of the proband), and III-1 (daughter of the proband). The variant site is indicated by red arrows. Double peaks at the variant position in I-1 and III-1 indicate heterozygous status (T/G), whereas single peaks in II-1 and II-2 indicate homozygosity (G/G). Roman numerals denote generations, and Arabic numerals denote individuals within each generation.

Because of the combination of early-onset bilateral nephrolithiasis, rapid progression to ESKD, and a positive family history, a hereditary stone disorder was suspected. Genetic testing identified a homozygous *AGXT* variant, NM_000030.2: c.740T>G (p.Leu247Arg), in the proband. Sanger sequencing was subsequently performed for familial validation (Figure 1).

**FIGURE 2:**
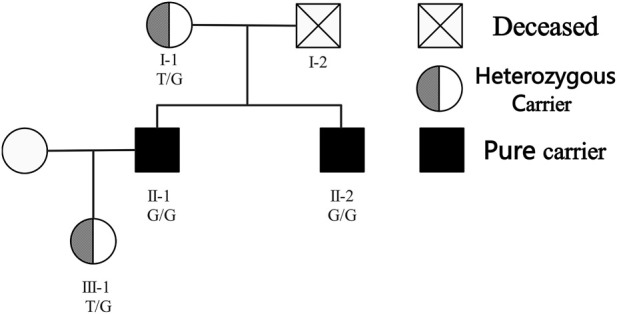
Pedigree of the family. Squares represent males and circles represent females; filled symbols indicate homozygotes, half-filled symbols indicate heterozygotes, and open symbols indicate unaffected individuals. A cross (×) within a symbol indicates individuals who were not genotyped.

Both the proband and his older brother were homozygous for the variant, whereas the proband’s mother and daughter were heterozygous carriers. This segregation pattern was consistent with autosomal recessive inheritance and supported the diagnosis of PH1 in the appropriate clinical context (Figure 2).

At present, this variant appears to be rare, and we were unable to identify a clearly documented prior clinical report specifically describing the same homozygous variant during our literature review. However, because no functional assays were performed, its pathogenic role should be interpreted in conjunction with the phenotype and segregation findings.

## 5 Follow-up and treatment

Both affected siblings progressed to ESKD and required long-term dialysis. At the maintenance dialysis stage, the proband was treated with hemodialysis alone, whereas his older brother received combined hemodialysis, hemofiltration, and hemoperfusion. During follow-up, the proband developed progressive extra-renal manifestations, including blindness, severe tooth loss, painful soft-tissue lesions, and extensive extra-skeletal deposition on imaging.

**FIGURE 3:**
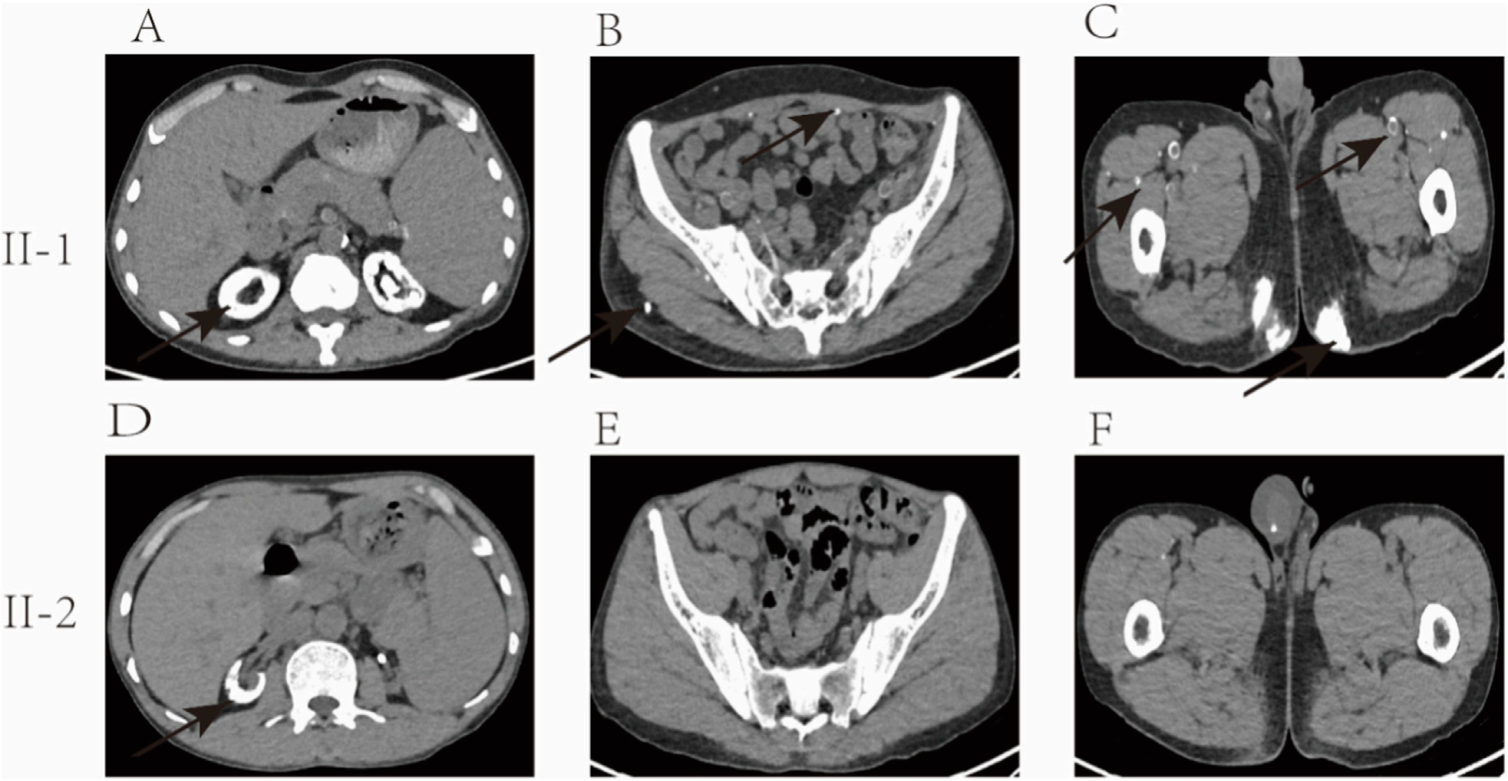
Radiological findings in the two affected siblings. **(A–C)** Axial computed tomography images of the proband (II-1), showing bilateral renal atrophy with diffuse calcification **(A)**, pelvic soft-tissue calcification **(B)**, and gluteal soft-tissue calcification **(C)**. **(D–F)** Corresponding computed tomography images of the brother (II-2), showing bilateral renal atrophy with diffuse calcification **(D)**, without apparent pelvic soft-tissue calcification **(E)** or gluteal soft-tissue calcification **(F)**. Arrows indicate the relevant abnormalities.

Repeat abdominal CT performed on 8 June 2025, demonstrated bilateral renal atrophy and widespread soft-tissue and marrow abnormalities. These findings were considered compatible with advanced systemic oxalosis, although contributions from chronic kidney disease-mineral and bone disorder (CKD-MBD) and long-term dialysis-related mineral metabolism abnormalities could not be excluded (Figure 3).

**FIGURE 4:**
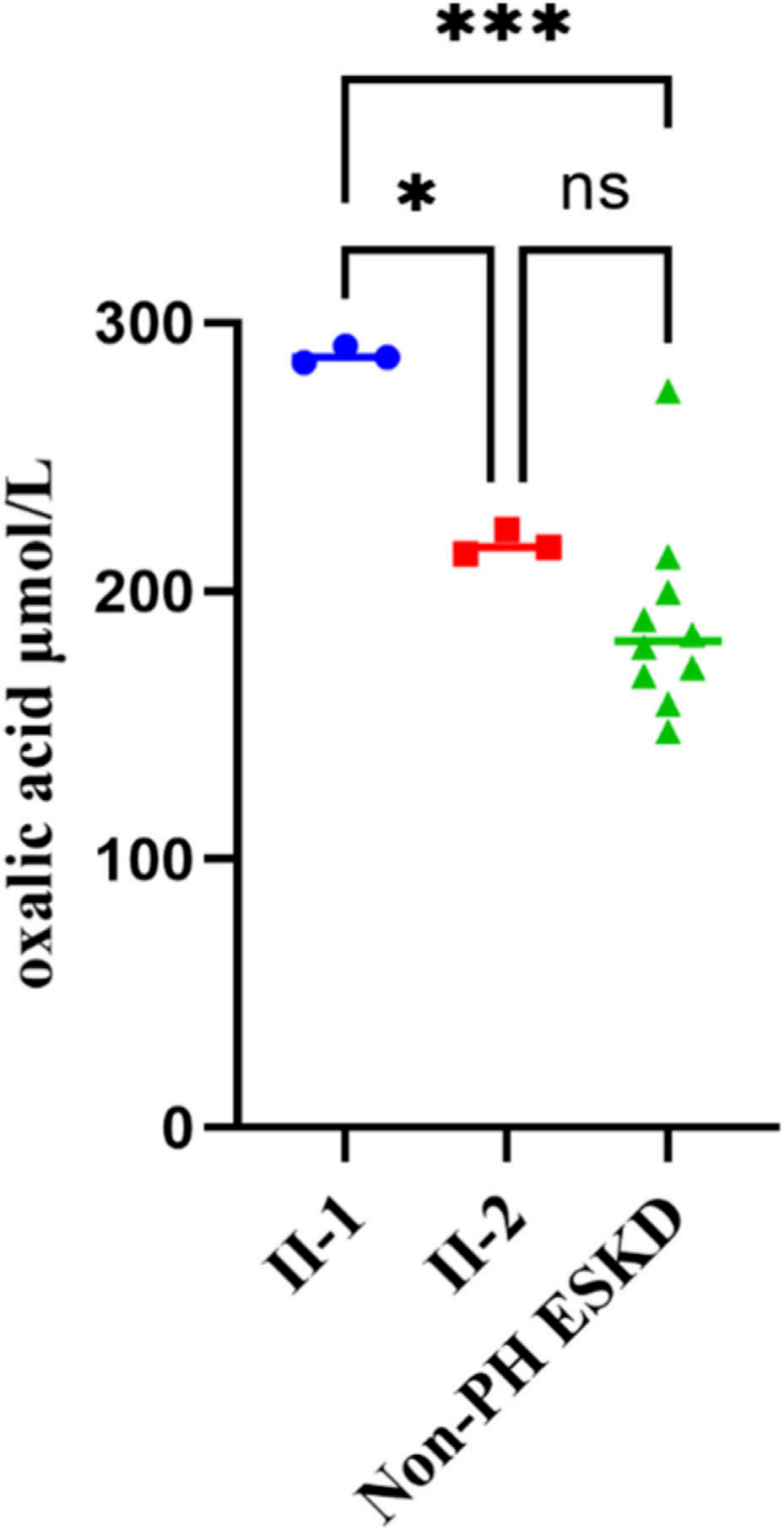
Comparison of plasma oxalate concentrations. Plasma oxalate levels in the proband (II-1) were markedly higher than those in his brother (II-2) and in patients with non–primary hyperoxaluria end-stage kidney disease (non-PH ESKD) receiving maintenance hemodialysis.

Peripheral blood samples obtained on 6 August 2025, were analyzed for plasma oxalate using the colorimetric oxalate assay kit (see Methods), revealing markedly higher levels in the proband than in his brother and non-PH ESKD controls (*n* = 15, *P* < 0.001 by one-way ANOVA) (Figure 4). In clinical practice within this family, the proband received maintenance hemodialysis alone, whereas his older brother underwent hemodialysis combined with hemofiltration and hemoperfusion. The older brother appeared to have better plasma oxalate control than the proband. These observations suggest that intensified blood purification may have a role as bridge therapy in advanced PH1, although this inference is based on observational family data rather than a formal comparative study.

At the latest follow-up, both patients remained on maintenance dialysis. Combined liver-kidney transplantation remained the definitive treatment option under consideration.

## 6 Discussion

This variant is located in a functionally important region of AGT and may affect protein conformation and enzymatic activity, although functional validation is lacking (Gatticchi et al., 2023). To our knowledge, this variant has not been clearly documented in the available literature and appears to be rare; however, its pathogenicity should be interpreted in conjunction with the clinical phenotype and segregation findings.

Regarding blood purification, the proband received maintenance hemodialysis alone, whereas his older brother received thrice-weekly hemodialysis (blood flow 300 mL/min) combined with post-dilution hemofiltration (replacement volume 23 L/session) and hemoperfusion using a HA130 resin adsorber, which enhanced oxalate clearance through diffusive, convective, and adsorptive mechanisms—consistent with prior reports demonstrating superior oxalate removal with multimodal extracorporeal therapy (Yamauchi et al., 2001). In this within-family observation, the older brother appeared to have better plasma oxalate control than the proband, suggesting that intensified extracorporeal blood purification may help reduce oxalate burden in advanced PH1. However, this finding should be interpreted cautiously because it was not derived from a formal comparative study. In terms of imaging manifestations of systemic oxalosis, the degree of subcutaneous and intermuscular calcification in this case greatly exceeded that typically reported in PH1. Particularly noteworthy were the rare complications of tooth loss and visual impairment in the proband, which further emphasize the aggressive phenotype associated with this variant. The ocular and dental manifestations observed in the proband extend beyond typical CKD-MBD or diabetic complications: retinal hemorrhages and vitreous opacities result from direct oxalate crystal deposition in retinal capillaries and vitreous humor, triggering endothelial apoptosis and pathological neovascularization—a hallmark of advanced PH1 (Michael et al., 2024). Similarly, progressive tooth loss stems from oxalate-mediated osteoclast activation and alveolar bone resorption, which is mechanistically distinct from periodontal destruction in diabetes (Hassona et al., 2024). These findings, when combined with severe extra-skeletal calcification, constitute a cohesive phenotype of aggressive systemic oxalosis driven by the c.740T>G variant.

This case also highlights a key diagnostic challenge in PH1: early manifestations are easily mistaken for more common disorders. Because the proband also had type 2 diabetes mellitus, his recurrent nephrolithiasis was initially attributed to diabetes-related metabolic abnormalities, which delayed consideration of a specific inherited etiology (Zhao et al., 2022). Previous studies have shown that early-onset disease (<40 years), bilateral multiple stones, nephrocalcinosis, and rapid renal function decline are important clues suggesting monogenic nephrolithiasis; both patients in this family met these characteristics (Schonauer et al., 2022). Therefore, for patients with early-onset, recurrent nephrolithiasis or nephrocalcinosis, even in the presence of common comorbidities such as diabetes, clinicians should maintain a high index of suspicion for inherited causes such as PH1 and pursue genetic testing promptly to avoid diagnostic delay.

From the perspectives of pathophysiology and therapeutic innovation, this case provides valuable practical experience in bridge therapy for end-stage PH1. Conventional dialysis has limited efficiency for oxalate removal, whereas the regimen used in the proband’s older brother—hemodialysis combined with hemofiltration and hemoperfusion—appeared to be associated with better plasma oxalate control than hemodialysis alone in the proband, potentially through enhanced diffusive, convective, and adsorptive clearance. This strategy may serve as an important bridge for patients who are not immediate candidates for combined liver-kidney transplantation. In addition, the c.740T>G variant may alter the conformation of the AGT catalytic center and thereby affect responsiveness to vitamin B6, suggesting the need for future genotype-guided individualized pharmacotherapy.

Regarding therapeutic strategies for advanced PH1, emerging RNA interference therapies such as lumasiran have demonstrated significant efficacy in lowering urinary and plasma oxalate in patients with preserved and advanced kidney function (Garrelfs et al., 2021b). For patients reaching ESKD, combined liver-kidney transplantation remains the only curative option, with optimal perioperative management requiring intensive oxalate-lowering strategies (Michae et al., 2023). The intensified blood purification regimen employed in the proband’s brother—combining hemodialysis, hemofiltration, and hemoperfusion—likely enhanced oxalate clearance through synergistic mechanisms, potentially serving as an effective bridge to transplantation in settings where novel therapies are inaccessible (Michael et al., 2024).

This report provides important insights into the clinical management of PH1, although several limitations should be acknowledged. First, the family sample size was small, and the father’s genotype was unavailable, limiting a comprehensive genetic assessment of this variant. Second, the observation period for treatment in the end-stage setting was relatively short, and long-term prognostic data require further follow-up. Notably, a multidisciplinary collaborative approach played a critical role in management of this family. Third, urinary oxalate excretion data were unavailable during the end-stage follow-up period due to logistical constraints, limiting comprehensive assessment of systemic oxalate burden. As emphasized in recent reviews on oxalate homeostasis, integrated evaluation of both plasma and urinary oxalate is ideal, though plasma levels remain informative in advanced kidney disease when renal excretion is minimal (Hassona et al., 2024).

## Data Availability

The datasets presented in this study can be found in online repositories. The names of the repository/repositories and accession number(s) can be found in the article/supplementary material.
